# Single‐cell analysis reveals cytotoxic and memory CD8^+^ T cells associated with prolonged survival in relapsed/refractory leukaemia patients after haplo+cord haematopoietic stem cell transplantation

**DOI:** 10.1002/ctm2.70529

**Published:** 2026-02-04

**Authors:** Hua Li, Zheyang Zhang, Ming Zhu, Xiaofan Li, Jinxian Dai, Ping Chen, Fei Chen, Xianling Chen, Yiding Yang, Xiaohong Yuan, Ronghan Tang, Zhijuan Zhu, Hongli Lin, Ting Lin, Mengsha Tong, Tao Chen, Yuanzhong Chen, Jialiang Huang, Nainong Li

**Affiliations:** ^1^ Hematopoietic Stem Cell Transplantation Center Fujian Institute of Hematology Fujian Provincial Key Laboratory on Hematology Department of Hematology Fujian Medical University Union Hospital, Gulou District Fuzhou China; ^2^ Department of Hematology Xiamen Medical College Affiliated Second Hospital, Jimei District Xiamen China; ^3^ State Key Laboratory of Cellular Stress Biology Xiang'an Hospital School of Life Sciences Faculty of Medicine and Life Sciences Xiamen University Xiamen Fujian China; ^4^ National Institute for Data Science in Health and Medicine Xiamen University Xiamen Fujian China; ^5^ Translational Medicine Center on Hematology Fujian Medical University Fuzhou China; ^6^ Department of hematology School of Medicine Xiamen University Xiamen Fujian China

**Keywords:** CD8^+^ T cells, haplo+cord HSCT, relapsed/refractory leukaemia, single cord HSCT, single‐cell multi‐omics

## Abstract

**Backgroud:**

Allogeneic haematopoietic stem cell transplantation (allo‐HSCT) is a curative treatment for haematological malignancies. Sequential transplantation of haploidentical stem cell and umbilical cord blood (haplo+cord HSCT) among recipients with relapsed/refractory (R/R) leukaemia exhibited superior survival outcomes compared with single cord HSCT. However, the underlying mechanisms remain unclear.

**Methods:**

Here, we profiled and compared single‐cell gene expression and chromatin accessibility in bone marrow from 16 patients receiving haplo+cord or single cord HSCT.

**Results:**

We observed distinct compositions and functions of global immune landscapes, with haplo+cord HSCT exhibiting effective anti‐tumour and anti‐viral immunity mediated by type I interferon signalling. Analysis of T cells revealed specific CD8^+^ T cell subtype (CD8‐c1), enriched in recipients with haplo+cord HSCT, which was also confirmed by flow cytometry. Functionally, gene signature scoring suggests a dual effector and memory property of CD8‐c1 that potentially offers long‐term protection. Furthermore, single‐cell multi‐omics analysis delineated the expression of cytotoxic‐related genes up‐regulated in CD8‐c1 are cooperatively regulated by enhancer networks. Notably, a proportion‐based survival analysis indicated that high proportion of CD8‐c1 was associated with better survival.

**Conclusion:**

Our results collectively demonstrate that a population of CD8^+^ T cells with effector and memory properties contributes to improved survival in patients with R/R leukaemia receiving haplo+cord HSCT.

## Graphical abstract

1. Haplo+cord HSCT yields superior survival compared with single cord HSCT in patients with R/R leukaemia.

2. Single‐cell RNA‐seq revealed a functionally critical CD8⁺ T cell population, with cytotoxic and memory phenotypes, which expands after haplo+cord HSCT and tracks with prolonged survival.

3. Multi‐omics profiling at single‐cell resolution showed that the CD8⁺ T cell elevated cytotoxic‐related genes is orchestrated by a cooperative enhancer network.

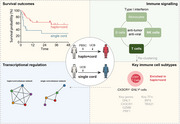

## 1 INTRODUCTION

Allogeneic haematopoietic stem cell transplantation (allo‐HSCT) is one of the most effective methods for curing haematological malignancies, particularly in patients with relapsed/refractory (R/R) leukaemia.^1^ The success of allo‐HSCT depends on the graft‐versus‐leukaemia (GVL) effect and the prevention of severe graft‐versus‐host disease (GVHD).^2^ Moreover, immune reconstitution in patients receiving allo‐HSCT relies on the ability of the donor graft for stem cell differentiation and development.^3^ For patients lacking human leukocyte antigen (HLA)‐matched sibling or unrelated donors, haematopoietic stem cells for transplantation can be derived from umbilical cord blood (UCB) and haploidentical donors.^4^, ^5^ UCB grafts have the natural immune advantage of a GVL effect and lower GVHD but are associated with delayed immune reconstitution, which can lead to increased risk of infections and early mortality.^6^, ^7^, ^8^ Conversely, haploidentical haematopoietic stem cell transplantation (haplo‐HSCT) has the advantage of rapid engraftment but is associated with a higher incidence of chronic GVHD.^9^, ^10^ To overcome these drawbacks while retaining the advantages, sequential transplantation of haploidentical stem cells and UCB stem cells (haplo+cord HSCT) has been proposed as an alternative, showing improved clinical outcomes in patients with R/R leukaemia.^11^, ^12^, ^13^ In a multi‐centre, randomised phase 3 trial, Zhou et al.^14^ reported that haplo+cord HCT significantly enhances overall survival (OS) and disease‐free survival (DFS) compared with haplo‐HSCT in patients with acute myeloid leukaemia. Our previous prospective clinical trial also found that haplo+cord HSCT improved the OS and DFS in patients with R/R leukaemia compared with single cord HSCT.^15^ Immune reconstitution following HSCT is a critical process for patient recovery and long‐term survival, involving the restoration of both innate and adaptive immune systems.^16^ Delays in Immune reconstitution significantly limit transplantation success, increasing risks of infection and relapse.^17^ Several studies have linked the clinical benefits of haplo+cord HSCT with T cell diversity, rapid natural killer (NK) and B cells reconstitution, rapid neutrophil and platelet recovery,^18^ as well as lower GVHD.^19^, ^20^ Adaptive immunity, involving B and T cells, requires 1–2 years for complete reconstitution.^21^ Because T‐cell recovery is the principal determinant of protection against viral infections and relapse,^22^ its kinetics after allo‐HSCT are intensively studied. The development of a new T‐cell repertoire is influenced by factors such as conditioning regimens, infections and GVHD.^23^ And quantitative haematopoietic recovery does not necessarily equate to functional immune competence.^24^ Consequently, post‐transplant immune reconstitution after haplo+cord HSCT, especially T‐cell recovery, remains largely undefined and poorly characterised.

Single‐cell techniques, such as single‐cell RNA‐sequencing (scRNA‐seq) and single‐cell assay for transposase‐accessible chromatin using sequencing (scATAC‐seq), facilitate the exploration of cellular heterogeneity and functionality during haematopoiesis and related diseases.^25^, ^26^, ^27^, ^28^, ^29^, ^30^ These approaches have been instrumental in delineating the dynamic immune reconstitution patterns following allo‐HSCT.^31^, ^32^ Additional studies identified distinct subsets of haematopoietic stem cells with dominant role in posttransplant haematopoietic reconstitution^33^ and the pathway to promotes haematopoietic reconstitution.^34^ However, the transcriptomic and epigenetic landscapes of R/R leukaemia after haplo+cord HSCT are still lacking, which impeded a comprehensive understanding of the mechanisms driving immune reconstitution.

Here, we collected BM samples from patients with R/R leukaemia who received haplo+cord or single cord HSCT and performed scRNA‐seq and scATAC‐seq on these samples. We found significant gene expression changes within each cell type between the two transplantation regimens, with haplo+cord derived cells involved in response to virus and type I interferon (IFN‐I) signalling pathway. In addition, we identified a subset of CD8^+^ T cells, with an increased cytotoxicity and memory signature score, enriched in haplo+cord HSCT and associated with improved survival. Taken together, our single‐cell multi‐omics analysis of post‐transplantation BM provided comprehensive insights into the underlying mechanisms of immune reconstitution in patients with R/R leukaemia after haplo+cord HSCT.

## 2 RESULTS

### 2.1 Haplo+cord HSCT improves survival of patients with R/R leukaemia

**FIGURE 1:**
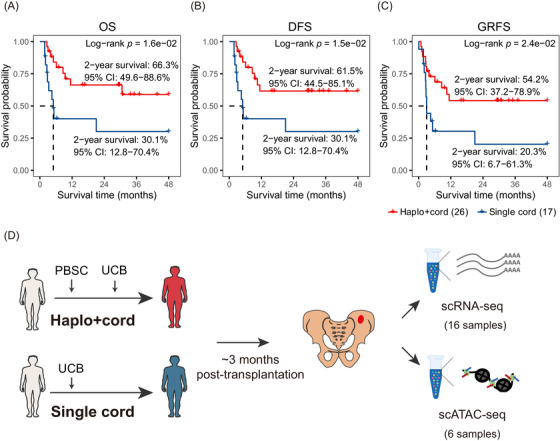
Comparison of overall survival outcomes of patients with R/R leukaemia receiving haplo+cord or single cord HSCT. (A–C) The Kaplan–Meier curves illustrate the overall survival (OS; A), disease‐free survival (DFS; B) and GVHD‐free, relapse‐free survival (GRFS; C) of 43 patients, categorised by transplantation strategies. *p* Values were calculated using the log‐rank test. (D) Schematic representation of single‐cell gene expression and chromatin accessibility profiling of bone marrow samples from post‐transplantation patients by using scRNA‐seq and scATAC‐seq. *Abbreviations*: PBSC, peripheral blood haematopoietic stem cells; UCB, umbilical cord blood stem cells.

To evaluate the clinical efficacy of the two transplantation strategies in the treatment of R/R leukaemia, we retrospectively compared the overall clinical outcomes of patients receiving haplo+cord HSCT or single cord HSCT (Figures 1 and S1). In total, we enrolled 43 patients with R/R leukaemia, among which 26 patients who received haplo+cord HSCT, whereas 17 who received single cord HSCT. The baseline clinical characteristics of patients and preconditioning regimens are shown in Table S1 and Figure S1A. We found that the median recovery time of neutrophil and platelet were 15.8 days (95% confidence interval (CI) 13.2–18.4) and 19.8 days (95% CI 15.4–24.3) in the haplo+cord group compared with 19.3 days (95% CI 17.7–20.9) and 33.5 days (95% CI 29.5–37.5) in the single cord group (log‐rank test, *p *< .05; Figure S1B,C). We next compared their survival outcomes and found that the haplo+cord group had superior survival outcomes with a 2‐year OS of 66.3% (95% CI 49.6–88.6%), DFS of 61.5% (95% CI 44.5–85.1%) and GRFS of 54.2% (95%CI 37.2–78.9%) compared with 30.1% (95% CI 12.8–70.4%), 30.1% (95% CI 12.8–70.4%) and 20.3% (95% CI 6.7–61.3%) in the single cord group (log‐rank test, *p < *.05; Figure 1A–C). Meanwhile, we did not detect significant differences in grade III–IV acute GVHD [12.5% (95% CI 0.8–25.8%) vs. 11.8% (95% CI 0–27.0%), log‐rank test, *p* = .681] and chronic GVHD [20.1% (95% CI 0–37.7%) vs. 37.5% (95% CI 0–79.2%), log‐rank test, *p* = .735] between the two groups (Figure S1D,E). Together, these results indicate that haplo+cord HSCT improves the survival outcomes of patients with R/R leukaemia.

### 2.2 Haplo+cord HSCT exhibits effective IFN‐I‐mediated anti‐tumour and anti‐viral immunity

**FIGURE 2:**
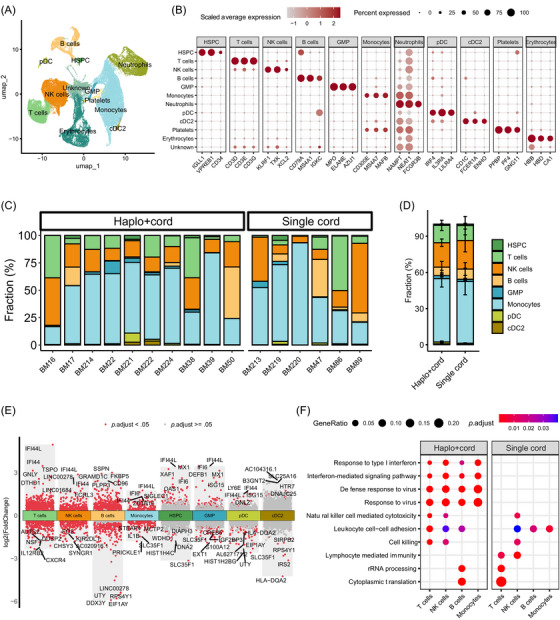
Global immune landscapes in bone marrow from patients receiving haplo+cord or single cord HSCT. (A) Uniform manifold approximation and projection (UMAP) of scRNA‐seq data from 16 patients coloured by the major cell types. (B) Expression of known marker genes for the major cell types identified in (A). (C) Bar plot showing the proportion of each cell type within each individual sample. (D) Bar plot showing the average proportion of each cell type in haplo+cord and single cord groups. Error bars are equal to the mean standard error (MSE) across individuals. (E) Differential gene expression between haplo+cord and single cord groups within each major cell type. Differentially expressed genes (DEGs; *p*.adjust < .05 and log2FoldChange > 0.25) are indicated in red, with labels for the top five genes. (F) Representative pathways enriched in DEGs for selected cell types in each transplantation strategy.

To understand the immune reconstitution in the BM microenvironment after transplantation, we collected post‐transplantation BM samples from 10 patients who received haplo+cord HSCT and six who received single cord HSCT and performed scRNA‐seq in each sample using the chromium platform (10x Genomics) (Figure 1D and Table S2). After quality control, we obtained the transcriptome profiles for 93 592 high‐quality BM mononuclear cells (56 089 from haplo+cord HSCT and 37 503 from single cord HSCT), with a median of 2914 detected genes and 7883 UMIs per cell, respectively (Figure S2A and Table S3). After batch effects correction, the graph‐based clustering analysis revealed 21 cell clusters (Figure S2B,C). Using known cell markers, we identified 11 major cell types (Figures 2A and S2D), namely, haematopoietic stem and progenitor cells (HSPCs) (expressing CD34 and *IGLL1*), T cells (expressing CD3D), NK cells (expressing *KLRF1*), B cells (expressing CD79A and *MS4A1*), granulocyte–monocyte progenitor (GMP) (expressing *MPO*), monocytes (expressing *MAFB*), neutrophils (expressing *NAMPT* and *NEAT*), plasmacytoid dendritic cells (pDC) (expressing *IRF4* and *IL3RA*), type 2 conventional dendritic cell (cDC2) (expressing *CD1C*), platelets (expressing *PPBP* and *PF4*), erythrocytes (expressing *HBB* and *HBD*) and a population of unknown cells with low expression of markers (Figure 2B). While the proportion of each cell type within a sample varied across individuals, the average proportions of these major cell types were comparable between the haplo+cord and single cord groups (Figures 2C,D and S2E).

To compare the overall immune characteristics between different transplantation strategies, we performed differential expression analysis between the haplo+cord and single cord groups within each major cell type (Figure 2E and Table S4). Four cell types (T cells, NK cells, B cells and monocytes) exhibited substantial transcriptome changes, as evidenced by a significant number of differentially expressed genes. Notably, IFN‐induced proteins (*IFI44*, *IFI44L* and *MX1*) were up‐regulated in various cell types after haplo+cord HSCT, which involve in pathways related to response to type I IFN (IFN‐I) signalling pathway and response to virus (Figure 2F). Additionally, genes up‐regulated in T cells derived from the haplo+cord group were involved in immune response and cell killing pathways, while those up‐regulated in the single cord group are associated with translation and ribosome‐related processes (Figure 2F). Together, these results indicate that patients receiving haplo+cord HSCT exhibit more effective anti‐tumour and anti‐viral immune responses mediated by the IFN‐I signalling pathway compared with those receiving the single cord HSCT.

Next, we aimed to determine the differences in proportions at the subtype level. By extracting and re‐clustering T cells, NK cells, B cells and monocytes into subtypes, we observed that, except for T cells (see the next section for details), there were no significant differences in proportions between the two transplantation strategies (Figure S3).

### 2.3 Haplo+cord HSCT augments cytotoxic and memory CD8^+^ T cells expressing *CX3CR1* and *GNLY*

**FIGURE 3:**
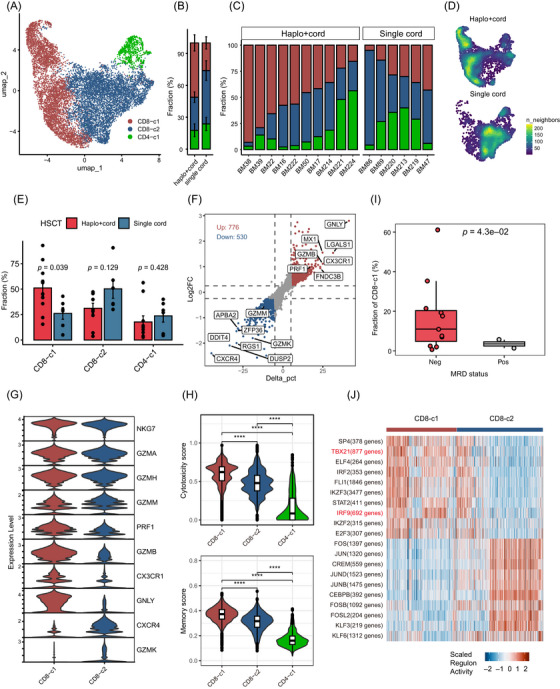
Reconstruction of T cells in bone marrow after haplo+cord and single cord HSCT. (A) UMAP of 11 575 T cells, identifying three clusters of T cell subtypes, as indicated by the labels, with CD8‐c1 and CD8‐c2 clusters defined by combinations of two key genes. (B) Bar plot showing the average proportion of each T cell subtype in haplo+cord and single cord groups. Error bars are equal to the MSE across individuals. (C) Bar plot showing the proportion of each T cell subtype within each individual sample. (D) UMAP plots of the density of T cells from patients receiving haplo+cord and single cord HSCT. (E) Proportion of each T cell subtype in haplo+cord and single cord groups. Error bars are equal to the MSE across individuals. Statistical analysis was performed using Wilcoxon signed‐rank test. (F) Scatter plot displaying gene expression changes between CD8‐c1 and CD8‐c2 subtypes. Each point represents a gene, with the *x*‐axis showing the difference in expression percentage (Delta_pct) and the *y*‐axis indicating the log_2_ fold change (log_2_FC). (G) Stacked violin plot showing the gene expression of immune‐related genes in two CD8^+^ T cell subtypes. (H) Violin plots showing the distribution of cytotoxicity scores and memory scores in each T cell subtype. T cell functional scores were calculated using AUCell with gene sets related to their function‐related genes (see section *Methods*). Statistical analysis was performed using Wilcoxon signed‐rank test. (I) Box plot showing the association between CD8‐c1 abundance and post‐transplant minimal residual disease levels. Statistical analysis was performed using Student's *t*‐test. (J) Heatmap of representative transcription factors (TFs) showing SCENIC estimated regulon activity for CD8^+^ T cells. Top 10 TFs with the highest difference in estimates of regulon activity in each CD8^+^ T cell subtype.

To further explore the different compositions and functions of T cells that mediate GVL effect^35^ between the haplo+cord and the single cord groups, we re‐clustered T cells into three subtypes, which included two groups of CD8^+^ T cells (with CD8‐c1 expressing *GNLY* and *CX3CR1*, and CD8‐c2 expressing *CXCR4* and *GZMK*), and a population of naïve CD4^+^ T cells (CD4‐c1; expressing *LTB*) (Figures 3A and S4A,B). By comparing the distributions of T cell subtypes between the two groups, we observed that the proportion of CD8‐c1 significantly increased in the haplo+cord group, accounting for 51.2% of T cells, compared with 26.1% in the single cord group (Figure 3B–E). Additionally, substantial transcriptome differences were detected between the two CD8^+^ T cell subtypes (Figure 3F). Genes up‐regulated in CD8‐c1 were enriched in pathways related to cell killing, chemokine signalling and IFN signalling (Figure S4C), while those in CD8‐c2 were linked to RNA processing (Figure S4D). We then conducted an examination of the expression of immune‐related genes and found high levels of *NKG7*, *GZMA*, *GZMH* and *GZMM* expression in the two CD8^+^ T cell subtype groups, indicating their identity as cytotoxic CD8^+^ T cells (Figure 3G). Notably, we observed that haplo+cord expanded CD8^+^ T cells (CD8‐c1) highly expressed the cytotoxic genes *GNLY, PRF1* and *GZMB*,^36^ as well as the memory CD8^+^ T cell‐specific chemokine receptor *CX3CR1*,^37^, ^38^ suggesting a dual effector and memory property for CD8^+^ T cells in the haplo+cord group (Figure 3G). In contrast, CD8‐c2 highly expressed the chemokine receptor *CXCR4*, which is related to the homing of progenitor cells to BM,^39^ and *GZMK*, a striking cellular hallmark of immune aging a murine model.^40^ Next, we quantified the cytotoxicity, memory, naïveness and exhaustion signature scores for each CD8^+^ T cell subtype based on the expression of function‐related genes.^41^ As expected, we found that two CD8^+^ T cell subtypes obtained relatively high scores for cytotoxicity and memory signatures, comparing with CD4‐c1 (Figure 3H), but not for naïveness or exhaustion signatures (Figure S4E). Notably, CD8‐c1 exhibited significantly higher cytotoxicity and memory scores compared with CD8‐c2. Furthermore, CD8‐c1 abundance is significantly associated with post‐transplant minimal residual disease negativity, thereby supporting this T‐cell phenotype to a superior GVL effect (Figure 3I).

To further explore the TFs that driving the distinct transcription program, we performed SCENIC analysis,^42^ which infers TFs by analysing the co‐expression of TFs and their putative target genes from scRNA‐seq data. We identified candidate TFs underlying the differences in gene expression in CD8‐c1, including IRF9 and TBX21 (Figure 3J). IRF9 was reported to prevent the exhaustion of CD8^+^ T cells during acute lymphocytic choriomeningitis virus infection,^43^ indicating that the activation of IRF9 may underlie the more effective immune responses of haplo+cord expanded CD8‐c1. Taken together, these data suggest that haplo+cord HSCT augmented the presence and function of effector and memory CD8^+^ T cells that highly express cytotoxic‐related genes.

### 2.4 Flow cytometry confirms the expansion of CD8‐c1 in recipients of haplo+cord HSCT

**FIGURE 4:**
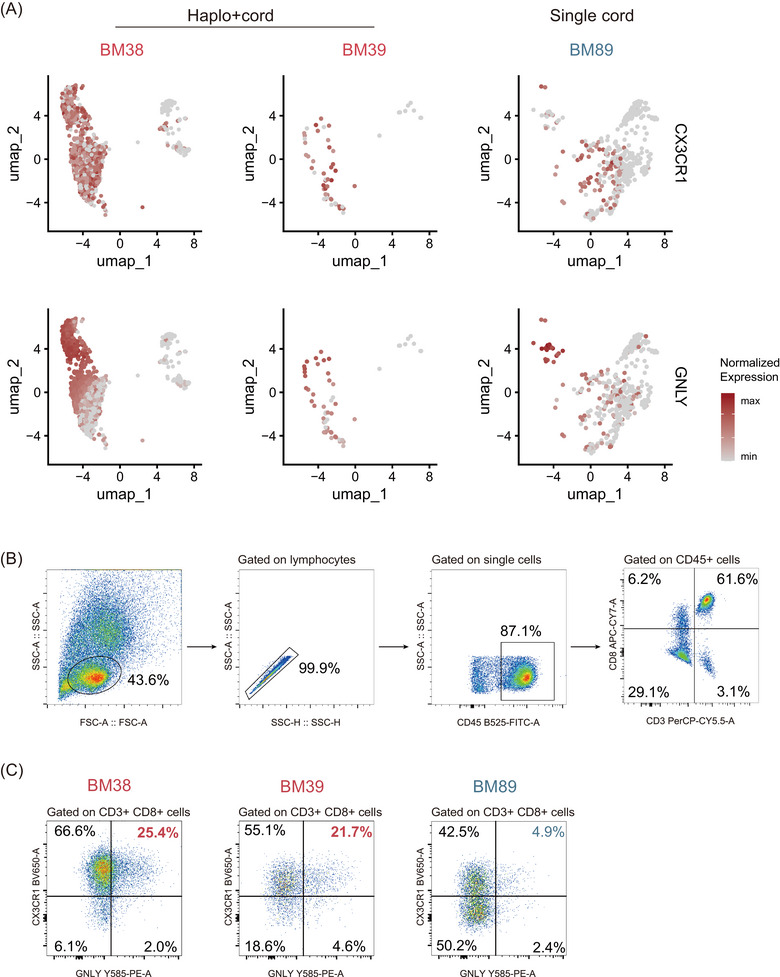
T cells distribution in representative patients revealed by scRNA‐seq and flow cytometry sorting strategy. (A) scRNA‐seq analysis revealed expression of *CX3CR1* (top) and *GNLY* (bottom) for T cells derived from two patients (BM38, BM39) receiving haplo+cord HSCT, and patient BM89 receiving single cord HSCT. (B) FACS gating strategy for sorting CD8^+^ T cells in BM samples. (C) FACS gating strategy for sorting *CX3CR1* ^+^ *GNLY* ^+^ CD8^+^ T cells from BM samples based on high CX3CR1 and GNLY expression.

To further confirm the expansion of CD8‐c1 in the haplo+cord group, we examined the expression of surface marker *CX3CR1* and the highly expressed gene *GNLY* in CD8^+^ T cells derived from individual patients. As expected, we found the haplo+cord derived T cells highly expressed *CX3CR1* and *GNLY* genes, compared with the single cord derived T cells (Figure 4A). Next, to validate scRNA‐seq analysis, we performed flow cytometry analysis on the residual BM samples from two recipients of haplo+cord HSCT (BM38, BM39) and one recipient of single cord HSCT (BM89) (Figure 4B,C). Notably, we observed a population of CD8^+^ T cells with co‐expression of CX3CR1 and GNLY enriched in the haplo+cord group (Figure 4C; 25.4 and 21.7% for BM38 and BM39 vs. 4.9% for BM89), further supporting the presence of CD8‐c1. Taken together, our flow cytometry analysis confirms the expansion of CD8‐c1 in recipients of haplo+cord HSCT.

### 2.5 Chromatin landscapes delineate lineage‐defining gene expression after HSCT

**FIGURE 5:**
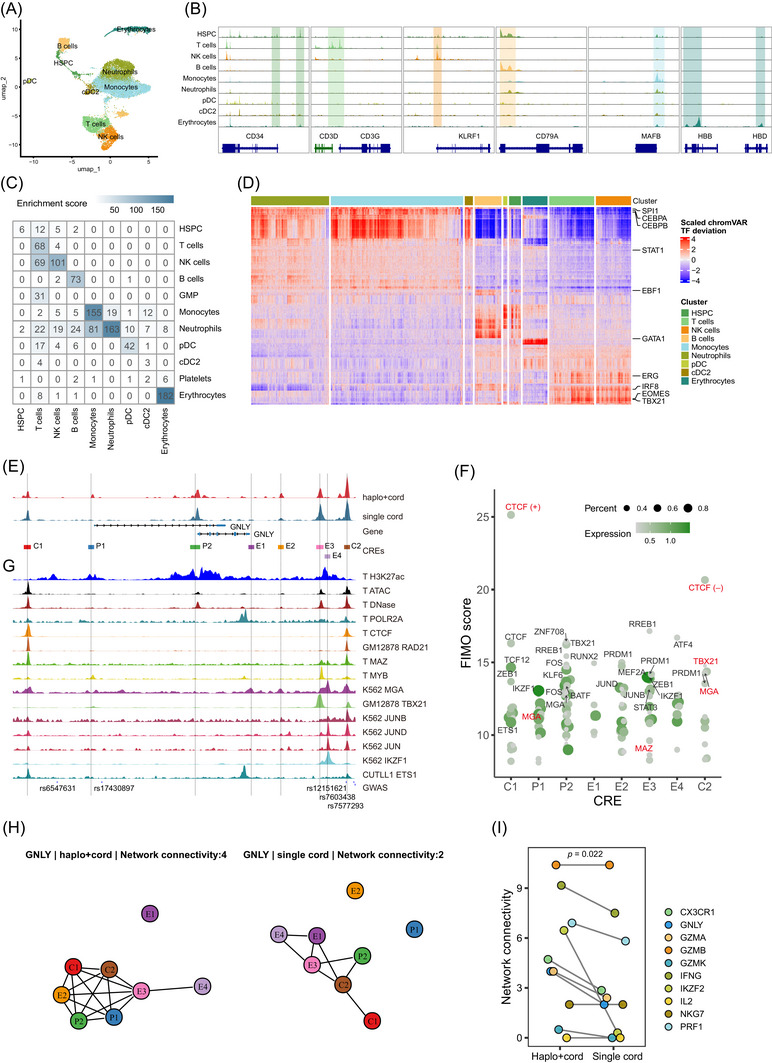
Single‐cell chromatin accessibility analysis revealed an enhancer cluster controlling *GNLY* expression in CD8^+^ T cells. (A) UMAP of scATAC‐seq data in bone marrow samples from six patients receiving haplo+cord or single cord HSCT, colour‐coded by the inferred major cell types. (B) Genome browser tracks showing aggregated chromatin accessibility of cells grouped by major cell types around the loci of marker genes. More marker genes for each cell type are shown in Figure S5B. (C) Single‐cell multi‐omics analysis revealed the enrichment of cell‐type‐specific genes (columns) in putative target genes of cell‐type‐specific cis‐regulatory elements (CREs) (rows). Enrichment score is defined as the −log_10_(hypergeometric test *p* values). (D) Heatmap of transcription factor (TF) motif accessibility *Z* scores (rows) in each cell (columns). The top 250 TF motifs, ranked by cell–cell variability, are shown, with representative TFs labelled. Major cell types defined in (A) are indicated in different colours. (E) Genome browser tracks showing the *GNLY* enhancer cluster. Aggregated chromatin accessibility of CD8^+^ T cells grouped by transplantation strategy are shown. (F) Prediction of TFs binding on individual CRE based on motif analysis and gene expression in CD8^+^ T cells derived from the the haplo+cord group. (+): motif on forward strand; (−): motif on reverse strand. TFs verified by ChIP‐seq data in T cells (E) are labelled by red colour. (G) ChIP‐seq of regulators binding on the *GNLY* enhancer cluster in blood cells. (H) eNet analysis revealed the transcriptional regulation network among eight CREs on the GNLY locus in CD8^+^ T cells derived from the haplo+cord and single cord groups. Each node represents a CRE, while each edge represents the potential interaction between two CREs predicted by eNet. (I) Comparison of network connectivity in cytotoxic‐related genes between the haplo+cord and single cord groups. Statistical analysis was performed using paired Student's *t*‐test.

To explore the gene regulation network driving distinct transcriptional programs of immune cells, we performed scATAC‐seq assays on six samples with matching scRNA‐seq, including three patients who received haplo+cord HSCT and three patients who received single cord HSCT, using the chromium platform (10× Genomics) (Figure 1D and Table S2). After quality control and batch correction, we obtained 15 121 cells for further analysis (6987 cells from the haplo+cord group and 8134 cells from the single cord group), with a total of 147 301 peaks and a median of 3365 fragments per cell (Figure S5A). We annotated the scATAC‐seq cell clusters as HSPC, T cells, NK cells, B cells, monocytes, neutrophils, pDC, cDC2 and erythrocytes by transferring the major cell type labels from scRNA‐seq data^44^ (Figure 5A). Consistent with scRNA‐seq data, we found that the major cell types showed cell‐type‐specific chromatin accessibility and imputed expression of known markers, including CD34, CD3D, *KLRF1*, CD79A, *MAFB*, *NAMPT*, *IRF4* and *HBB* (Figures 5B and S5B,C). In total, using differential accessible peak analysis we identified 37 876 cell‐type‐specific cis‐regulatory elements (CREs) (Figure S5D). We observed a high correlation between the cell‐type‐specific chromatin accessibility of CREs and the expression of their putative target genes (Figure 5C), suggesting their regulatory roles in controlling the expression of lineage‐defining genes of immune reconstitution after transplantation. In addition, we applied ChromVAR^45^ to calculate the activity scores of TF motifs for each cell and found that they exhibited high variance across all cells representing known master regulators of haematopoiesis, such as CEBPD, ATF4 and RUNX1 (Figure S5E). Hierarchical clustering of single‐cell TF *Z*‐scores identified cell‐type‐specific TF motifs (Figure 5D), such as, TBX21 (also known as T‐bet) for T and NK cells,^46^ EBF1 for B cell,^47^ as well as GATA1 and CEBPB for erythroid and myeloid development.^48^ Overall, single‐cell chromatin accessibility profiling provides a high‐quality resource for exploring the underlying gene regulation in immune cells after transplantation.

### 2.6 *GNLY* expression in CD8^+^ T cells is regulated by CTCF‐mediated chromatin loop

CREs play important roles in regulating gene expression.^49^ We used the *GNLY* gene, with the highest expression in CD8‐c1, as an example to explore the gene regulation networks controlling the differential transcriptional programs between the two transplantation strategies.

Using our scATAC‐seq data, we identified eight CREs in the *GNLY* locus, named C1, C2, P1, P2 and E1–4 (Figure 5E). To interrogate TF occupancy at each CRE, we considered both motif occurrences by Find Individual Motif Occurrences (FIMO) analysis^50^ and the expression of TFs in CD8^+^ T cells in the haplo+cord group (Figure 5F). Among these CREs, P1 and P2 were the alternative promoters of *GNLY* transcripts, whereas C1 and C2 were CTCF‐binding sites with convergent orientation CTCF motif, which was verified by the CTCF ChIP‐seq data in T lymphocytes (Figure 5G and Table S5). In addition, we detected that MYC‐associated zinc finger protein (MAZ) and RAD21 (a well‐known cohesin subunit) bound on C1 and C2 in T‐lymphocytes and the GM12878 cell line, respectively (Figure 5G). These data suggested the formation of a chromatin loop on C1 and C2 anchors that is mediated by CTCF, MAZ and cohesin.^51^, ^52^ We found that the C2 CTCF‐binding site contained several GWAS SNPs, such as rs12151621, rs7603438 and rs7577293 (Figure 5G), which have been reported to be associated with *GNLY* expression in human blood plasma proteome GWAS studies.^53^, ^54^, ^55^ This further illustrated the importance of the CTCF‐mediated chromatin loop for the expression of *GNLY*. Applying our recently developed algorithm eNet,^56^ we built enhancer networks controlling *GNLY* expression in CD8^+^ T cells. eNet confirmed the potential interaction between C1 and C2, as well as both P1 and P2 interacting with C1, C2, E2, E3 and E4 (Figure 5H). Interestingly, while these CREs in the *GNLY* locus were indistinguishable in their chromatin accessibility between the haplo+cord and single cord groups (Figure 5E), we observed *GNLY* enhancer networks was more complex in the haplo+cord group than in the single cord group (Figure 5H). Interestingly, our previous work suggested the complexity of enhancer networks are associated with the expression of cell identity and disease genes.^56^ This pattern was also observed for several cytotoxic‐related genes that were highly expressed in haplo+cord derived T cells (Figures 5I and S5F–H). Taken together, these results delineate that an enhancer network within the CTCF‐mediated chromatin loop controls the expression of *GNLY* in CD8^+^ T cells reconstructed from haplo+cord HSCT.

### 2.7 In silico cytometry analysis reveals the prognostic value of CD8^+^ T cell subtypes

**FIGURE 6:**
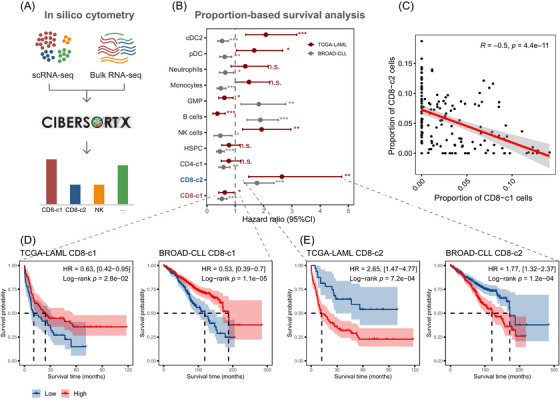
The prognostic value of CD8^+^ T cell subtypes through in silico cytometry analysis. (A) The diagram of in silico cytometry by CIBERSORTx. (B) Forest plot showing the association between cell type proportion and overall survival in the two available RNA‐seq datasets. (C) Scatter plot showing the correlation between cellular proportions of CD8‐c1 and CD8‐c2 subtypes across samples in TCGA‐LAML. Correlation coefficients and *p* value are computed using Spearman method. (D and E) The Kaplan–Meier curves illustrating the association of CD8‐c1 (D) and CD8‐c2 (E) proportion with overall survival on the TCGA‐LAML and BROAD‐CLL cohort. The optimal cut point for stratifying high‐ and low‐level infiltration was obtained by using the maximally selected rank statistics. High infiltration in red, low infiltration in blue. *p* values were calculated using the log‐rank test.

To characterise the influence of the proportion of CD8^+^ T cell subtypes on the prognosis of leukaemia, we adopted in silico cytometry analysis (Figure 6A) by querying publicly available acute myeloid leukaemia cohort on The Cancer Genome Atlas (TCGA‐LAML)^57^ and chronic lymphocytic leukaemia cohort on Broad institute (BROAD‐CLL).^58^ Strikingly, CD8‐c1 and CD8‐c2 exhibited a significantly consistent prognostic trend in their proportion‐based survival analysis (Figure 6B). Notably, the proportions of CD8‐c1 and CD8‐c2 were negatively correlated in both cohorts (Figures 6C and S6A). Higher infiltration of CD8‐c1 was associated with better prognosis in leukaemia patients, whereas CD8‐c2 showed the opposite trend (Figures 6D,E and S6B). These findings strengthen the notion that CD8‐c1 may contribute to prolonged survival in patients with R/R leukaemia undergoing haplo+cord HSCT.

## 3 DISCUSSION

Recent studies, including ours, have shown that haplo+cord HSCT, which combines the advantages of UCB and haploidentical grafts, can significantly improve the survival of patients with R/R leukaemia.^11^, ^12^, ^13^, ^15^ Here, our single‐cell analysis revealed distinct compositions and functions of global immune landscapes in post‐transplantation BM. Overall, the haplo+cord group exhibited effective anti‐tumour and anti‐viral immunity mediated by IFN‐I signalling. CD8^+^ T cells are actively involved in the GVL effect. CD8^+^ T cells recovery is typically observed within 100 days to 6 months post‐HSCT.^59^ The effective reconstitution of CD8^+^ T cells has been shown to correlate with a reduced incidence of leukaemic relapse and NRM, as well as improved survival outcomes.^60^, ^61^ Robust immune reconstitution, particularly of CD8⁺ T cells after HSCT is associated with superior clinical outcomes, enabling control of infection and mediating the GVL effect.^62^ Studies have consistently linked robust CD8⁺ T‐cell reconstitution following haploidentical stem‐cell transplantation to reduced relapse^63^ and improved survival benefit in high‐risk acute leukaemia.^64^ These cytotoxic lymphocytes are also playing a central role in restoring antiviral immunity post‐transplant.^65^

Our single‐cell sequencing data, sourced from patients within 3 months post‐transplantation, reveals mature CD8^+^ T cells. This indicates these cells play a key role in the early GVL effect after haplo+cord HCST, boosting patient survival. We identified two CD8^+^ T cell subsets, CD8‐c1 in the haplo+cord group and CD8‐c2 in the single cord group. Differential expression analysis revealed that CD8‐c1 had an increased expression of cytotoxicity and memory genes (*GNLY*, *CX3CR1*, *PRF1* and *GZMB*), indicating the potential role in exerting significant anti‐viral and anti‐tumour immune activity.^38^, ^66^, ^67^ Our proportion‐based survival analysis further demonstrated the relationship between CD8‐c1 and prolonged survival in patients with leukaemia, but further validation is needed to determine its prognostic significance in post‐HSCT settings.

Preconditioning regimen plays an important role in immune reconstitution. Post‐transplant cyclophosphamide (PTCy) is currently considered a highly effective strategy for preventing both acute and chronic GvHD. In our haplo+cord HSCT preconditioning protocol, cyclophosphamide (Cy) was added between haplo‐HSCT and single cord HSCT for GVHD prophylaxis (Figure S1A). Early T‐cell reconstitution relies on the expansion of donor‐derived mature T cells, whereas complete T‐cell reconstitution requires de novo production of naïve T cells through thymopoiesis.^68^ Roberto et al.^69^ showed that nearly all donor T cells with proliferation markers were vulnerable to Cy after haplo‐HSCT. Cieri et al.^70^ revealed that the majority of T cells within the first 30–90 days after HSCT under PTCy conditions originated from the donor naïve compartment. A key difference between the two transplant strategies compared in this study is the use of PTCy for GVHD prophylaxis in the haplo+cord cohort. It is well established that PTCy selectively depletes rapid proliferating alloreactive T cells in the early post‐transplant period, while sparing quiescent immune subsets such as naïve T cells and stem cell memory T cells (T_SCM_).^69^, ^71^ This presentation creates a permissive niche that supports robust de novo T‐cell reconstitution and differentiation of long‐lived memory populations.^72^ Our single‐cell data reveal a significant expansion of the cytotoxic CD8‐c1 subset in haplo+cord recipients. We therefore hypothesise that PTCy‐mediated landscape is a pivotal factor shaping this distinct immune profile. By eliminating alloreactive and proliferating T cells derived from the haploidentical donor, PTCy may facilitate the preferential expansion and differentiation of TSCM and naïve T cells into cytotoxic CD8‐c1 cells, thereby contributing to the potent GVL efficacy observed in the haplo+cord cohort. In contrast, the single cord cohort, which lacks PTCy, may not provide the same selective pressure, potentially leading to a less focused and effective cytotoxic T‐cell response. This proposed mechanism, lining PTCy to the chromatin and transcriptional landscape of recovering T cells, offers a plausible explanation for the superior clinical outcomes and merits further investigation in prospective studies. Granulysin (GNLY), which is secreted by cytotoxic CD8^+^ T cells, plays an important role in killing leukaemic cells. *GNLY* was highly expressed in CD8‐c1, consistent with the differences in enhancer profiles, in that some novel enhancer signals appeared near the *GNLY* gene. A recent study defined a distally accessible chromatin region downstream of *GNLY* in T cells.^73^ Going beyond this study, we further revealed that *GNLY* expression was controlled by a CTCF‐mediated chromatin loop involving multiple TFs, such as TBX21 and ETS1. ETS1 plays an essential role in NK cell cytotoxicity and production of IFN‐γ,^74^ providing clues for the transcriptional regulation of *GNLY* by ETS1 in T cells. Thus, our single‐cell multi‐omics analysis revealed the underlying mechanism of transcriptional regulation in the haplo+cord group.

Our study was subject to several limitations. First, our single‐cell data were constrained by a limited sample size, which restricted our ability to fully elucidate the underlying mechanisms. Second, the data were derived from a single‐centre study. And there is a lack of comparison with a contemporaneous cohort of patients receiving haploidentical HSCT. Thus, the generalisation of our findings would require validation through multi‐centre collaboration and the analysis of larger sample sizes. Third, our assessment was limited to a single time point post‐transplantation, which precluded a comprehensive analysis of immune reconstitution dynamics over time. Finally, all BM samples selected for scRNA‐seq/scATAC‐seq were obtained from patients who were alive at 3 months post‐transplant, which may introduce a selection bias toward a more favourable prognosis group. Despite these limitations, this study nonetheless represents a significant step toward understanding the mechanisms of HSCT at the single‐cell level.

## 4 METHODS

### 4.1 Study design, endpoints and definitions

This study was designed to retrospectively compare the overall clinical outcomes between patients undergoing two different transplantation strategies. The primary endpoint was DFS, whereas the secondary endpoints were OS, engraftment kinetics and GVHD. GRFS was defined as a composite endpoint comprising any death, relapse, grade III–IV acute GVHD or chronic GVHD. Neutrophil recovery was defined as the first of 3 consecutive days with an absolute neutrophil count exceeding 500/µL. Platelet recovery was defined as the first of 3 consecutive days with an absolute platelet count of 20 000/µL without transfusion. Primary graft failure was considered when a patient did not achieve neutrophil recovery by day 30 post‐transplantation, in the absence of bone marrow disease. Secondary graft failure was considered to occur when at least two haematopoietic cell lines were lost after initial haematopoietic recovery. Donor chimerism was analysed by STR‐PCR at the time of engraftment and at 3, 6 and 12 months, as well as annually after transplantation. GVHD was diagnosed and graded according to classic definitions: acute if it appeared before day 100 post‐infusion or chronic if it appeared after day 100 post‐infusion. DFS was defined as the time from transplantation to relapse or death from any cause.

### 4.2 Conditioning regimen, GVHD prophylaxis and stem cell infusion

The myeloablative condition regimen for all recipients was conducted as following (Figure S1): (1) fludarabine (25 mg/m^2^, iv, daily), cytarabine (2 g/m^2^, iv, daily) from day −13 through −9; (2) Cy (1.8 g/m^2^, iv, daily) on days −8 and −7, with mesna (2.5 g/m^2^) being administered to prevent Cy‐induced early bladder haemorrhage; (3) busulfan (Bu, 0.8 mg/kg, iv, q6h) from day −6 through −4, with phenytoin sodium being administered to prevent Bu‐induced seizures; (4) MCCNU (250 mg/m^2^, po) on day −3 to prevent central nervous system leukaemia. Peripheral blood stem cells were transfused on day 0, whereas UCB stem cells were transfused on day 6 in haplo+cord HSCT. UCB stem cells were transfused on day 0 in single cord HSCT.

For GVHD prophylaxis, patients in the haplo+cord group received low doses of anti‐thymocyte globulin (ATG), PTCy, cyclosporine A (CsA) and mycophenolate mofetil (MMF) (Figure S1A): (1) ATG (total dose 5 mg/kg, iv) on days −5 and −4. (2) Cy (1.8 g/m^2^, iv) on days +3 and +4. (3) CsA (3.0 mg/kg/day, iv) was administered from day +5 until stem cells were engrafted. Subsequently, CsA (targeting a blood concentration range of 200–300 ng/mL) was administered orally for 3 months. The CsA dose was reduced to 5–10% every week for 6 months and then fully stopped. If the patient was intolerant to CsA, administration of tacrolimus was adjusted to maintain a concentration of 5–15 ng/mL until day 180. (4) MMF (1.0 g/d) was administered from day +5 until engraftment of stem cells. Then, MMF was reduced to 0.5 g/d for 1 month and finally stopped. In the case of single cord HSCT, all patients received a low dose of ATG, CsA and MMF: (1) ATG (total dose 5 mg/kg, iv) on days −5 and −4. (2) CsA (3.0 mg/kg/day, iv) was administered from day −7 until stem cell engraftment. Subsequently, CsA was administered orally, with the CsA blood level and dosage reduction being consistent with those in the haplo+cord group. (3) MMF (1.0 g/d) was provided after UCB infusion on day 0 of stem cell engraftment; Then, MMF was reduced to 0.5 g/d for 1 month and fully stopped.

### 4.3 Retrospective survival analysis

This retrospective clinical trial was conducted at the Hemopoietic Stem Cell Transplantation Center, Fujian Medical University Union Hospital between January 2016 and December 2021. In total, we enrolled 43 patients with R/R leukaemia, including nine newly reported cases and 34 cases reported in our previous prospective clinical trial.^15^ Among these patients, 26 received haplo+cord HSCT, whereas 17 received single cord HSCT. All probabilities were given at 2‐years and provided with their 95% CI. The Kaplan–Meier method with the log‐rank test was used to estimate the probabilities of OS, DFS and GRFS. A *p *< .05 was considered statistically significant. The cumulative incidence of GVHD was calculated using competing‐risks analysis. Statistical analyses were performed using R package survival (version 3.5.8).

### 4.4 Clinical samples

To investigate the molecular and cellular mechanisms that underlie clinical outcomes, we collected BM samples approximately 3 months after transplantation to obtain fully chimeric BM. BM samples were obtained from 10 patients who received haplo+cord HSCT and 6 who received single cord HSCT. The baseline characteristics of patients are shown in Table S2.

### 4.5 Preparation and sequencing of scRNA‐seq library

BM samples were diluted 1:1 with cold PBS and centrifuged on Ficoll (GE 17‐1440‐02) to isolate BM mononuclear cells. Residual erythrocytes were removed using erythrocyte lysate (TIANGEN, RT122), washed twice and resuspended in cold PBS containing 0.04% BSA (Sigma; B2064). As the residue of mature erythrocytes in the bone marrow was difficult to be completely lysed in the experiments, we excluded erythrocytes in the downstream analysis. Single‐cell suspensions were filtered using 40 µm cell strainers. The process of cell preparation before loading onto the 10× chromium controller was less than 2 h. Trypan blue (0.2%) staining was used for the evaluation of cell numbers and viability under a microscope. Samples with cell viabilities >90% were used for sequencing. Libraries were constructed using the Single Cell 3′ Library Kit V3.1 (10× Genomics, Pleasanton, CA, USA). The transcriptome profiles of individual cells were determined using 10× genomic‐based droplet sequencing. Once prepared, indexed cDNA libraries were sequenced with paired‐end reads using an Illumina NovaSeq 6000 (Illumina, San Diego, CA, USA).

### 4.6 Preparation and sequencing of scATAC‐seq library

Single‐cell suspensions were obtained for the scATAC‐seq assay, as described above. Single cells were lysed with lysis buffer on ice for 3 min and washed once with wash buffer.^75^ Nuclei were resuspended in PBS containing 0.04% BSA, and approximately 10 000 nuclei were transposed and loaded onto the chip according to the protocol of the 10× Chromium Single Cell ATAC Reagent Kit v1.1. Libraries were sequenced on an Illumina NovaSeq 6000 using the following read lengths: read 1: 50, read 2: 50, i7 index: 8 and i5 index: 16.

### 4.7 Flow cytometry analysis

Single cell suspensions were prepared as describes above. One million cells were stained with CX3CR1 (Biolegend; 341625), CD8 (Biolegend; 344745), CD3 (Biolegend; 300327) and CD45 (Biolegend; 368508) in cell stain buffer for 30 min on ice. Cells were then washed twice with cell stain buffer (Biolegend; 420201) and resuspended in 100 µL cell stain buffer. To fix cells, 200 µL IC fixation buffer (eBioscience; 00‐8222‐49) were added to cells, kept at RT for 1 h. Fixed cells were washed with permeabilisation buffer (eBioscience; 00‐8333‐56) and stained with GNLY (Biolegend; 348003) at RT for 30 min. Stained cells were washed twice with permeabilisation buffer and resuspended in cell stain buffer for FACS analysis (Beckman, Cytoflex LX).

### 4.8 scRNA‐seq data processing

Raw sequence data from each sample were mapped to the human genome (build hg38) using CellRanger (version 7.2.0) provided by 10× Genomics. Using the preliminary clustering information for each sample, ambient RNA contamination was estimated and subsequently removed using the SoupX package (version 1.6.2).^76^ Raw gene expression matrices were then combined in R (version 4.3.2) and converted to a Seurat object using the Seurat R package (version 5.0.3).^77^ To ensure data quality, we removed cells that had either less than 1000 or more than 25 000 unique molecular identifiers (UMIs), less than 500 or more than 5000 expressed genes or over 10% UMIs derived from the mitochondrial genome. We used the remaining cells to exclude genes detected in less than 0.1% of total cells. For the remaining 93 592 cells, we used the ‘SCTransform’ function,^78^ which models the UMI counts using a regularised negative binomial model to remove the variation of sequencing depth, to normalise gene expression and regress out mitochondrial read count with the following parameters: vst.flavor = ‘v2’ and vars.to.regress = ‘percent.mt’. To exclude potential batch effects among different time points during library construction (Table S3), we performed batch effects correction, following the standard pipeline for dataset integration in Seurat, using library construction time points to split datasets (Figure S2B).

### 4.9 Dimension reduction and unsupervised clustering for scRNA‐seq data

To reduce the dimensionality of this dataset, the resulting 3000 variably expressed genes from the default setting were summarised using principal component analysis (PCA), and the first 30 principal components were further summarised using Uniform Manifold Approximation and Projection (UMAP) dimensionality reduction with the default settings of the ‘RunUAMP’ function. Then, ‘FindNeighbors’ and ‘FindClusters’ functions were used to calculate the neighbours among each cell and identify cell clusters.

### 4.10 scRNA‐seq cell annotation

After the first‐round of unsupervised clustering, we annotated each cell cluster according to canonical immune cell markers and identified the major cell types, including HSPCs, T cells, NK cells, B cells, GMP, monocytes, neutrophils, pDC, cDC2, platelets and erythrocytes. Three Seurat clusters (4, 18 and 20) with low expression of markers were labelled as #x02018;Unknown#x02019;. We excluded several cell types (erythrocytes, neutrophils and platelets) and unknown cells from downstream analysis due to lack of interest.

The procedure of the second‐round clustering for each major immune cell type was similar to that of the first‐round clustering, both of which started from raw expression matrices, then normalised gene expression, corrected batch effects, calculated PCA matrix and were then subjected to dimensionality reduction for visualisation. All analyses were performed using Seurat. Marker genes were detected using the ‘FindAllMarkers’ function.

### 4.11 scATAC‐seq data processing

scATAC‐seq raw sequence data were mapped to the human genome (build hg38) using CellRanger ATAC (version 2.1.0), generating a count matrix for open chromatin regions in single cells. Downstream analyses were performed using the Signac workflow (version 1.12.0),^79^ an extension of Seurat for the analysis, interpretation and exploration of single‐cell chromatin datasets. Briefly, the peak count matrix was combined and converted into a Seurat object using Signac. Genomic ranges and gene information were annotated for each peak. Subsequently, a series of additional quality control metrics, including the total number of fragments in peaks, fraction of fragments in peaks, ratio of reads in genomic blacklist regions, nucleosome banding signal and transcriptional start site enrichment score, were calculated for each single cell. High‐quality cells with a number of fragments in peaks greater than 1000 and less than 10 000, the fraction of fragments in peaks greater than 15%, reads in genomic blacklist regions less than 5%, nucleosome banding signals less than 4 and transcriptional start site enrichment scores larger than 4 were retained for downstream analyses.

### 4.12 Dimension reduction and unsupervised clustering for scATAC‐seq data

Latent semantic indexing (LSI) was applied to the high‐quality peak count matrix for downstream analyses as previously described.^80^ Term frequency‐inverse document frequency (TF‐IDF) normalisation was performed to correct for differences in sequencing depths across cells and peaks. The top 95% of peaks were then selected using the ‘FindTopFeatures’ function, followed by dimensional reduction using singular value decomposition on the TF‐IDF matrix. After LSI, we used the RunHarmony function in the Harmony R package (version 1.2.0)^81^ for integration with group.by.vars = 'Sample'. After correlating harmony components with sequencing depth using the ‘DepthCor’ function, we removed the first harmony component because of its correlation with sequencing depth rather than biological variation. Hence, 2 to 30 harmony components were used for non‐linear dimension reduction of UMAP projections to visualise cell clusters in a two‐dimensional space. Finally, the same harmony components were applied using the ‘FindNeighbors’ function to calculate the neighbours among each cell, and cell clusters were identified using the ‘FindClusters’ function.

### 4.13 scATAC‐seq cell annotation

We employed the AtacAnnoR R package (version 0.3.5)^44^ to annotate scATAC‐seq data using a two‐round annotation method with well‐annotated scRNA‐seq data as reference. In the first round, ATACAnnoR compared the gene activity profile derived from scATAC‐seq data with the reference gene expression profile, assigning reference cell type labels to a subset of the queried cells. In the second round, ATACAnnoR used a meta‐program matrix derived from genome‐wide ATAC peaks to predict the labels of the remaining query cells. We subsequent expanding these classifications to similar cells to generate a set of cluster‐extended assignments. The ChromVAR^45^ procedure was run with the ‘RunChromVAR’ function, using the JASPAR2020^82^ motif set to calculate the enrichment of chromatin accessibility at different transcription factors (TFs) motif sequences in single cells.

### 4.14 SCENIC analysis

To infer potential TFs from scRNA‐seq data, we applied pySCENIC (version 0.12.1),^42^, ^83^ which is a lightning‐fast python implementation of the SCENIC pipeline. The input matrix was the normalised expression matrix from Seurat and was further filtered according to the criteria recommended by pySCENIC.

### 4.15 Differential gene expression analysis

To identify marker genes for each cell type, we applied the ‘FindAllMarkers’ function with the parameter only.pos = ‘TRUE’. To compare transcriptomic differences between cell groups (e.g., CD8^+^ T cell subtypes), we calculated the log2 fold change between two groups and the percentage of cells where the gene was detected in each cell subtype using the ‘FindMarkers’ function. Significance of the difference was determined using the Wilcoxon Rank Sum test with Bonferroni correction. Differentially expressed genes (DEGs) were selected based on the statistical threshold (difference of the percentage of cells where the gene was detected between cell subtypes larger than 5%, log2 fold change larger than 0.25 and adjusted *p* < .05).

### 4.16 Pathway enrichment analysis of differentially expressed genes

The DEGs were categorised according to the functional gene sets in the pathway from The Molecular Signatures Database (MSigDB)^84^ using the clusterProfiler R package (version 4.8.1)^85^ for annotation and integrated discovery pathway enrichment analysis.

### 4.17 Definition of signature scores

To evaluate cell status at the signature level, we applied the AUCell R package (version 1.22.0)^42^ to calculate the signature score of single cells. Signature genes were obtained from published literature. For CD8^+^ T cells, naïveness scores were calculated using the following genes: *CCR7*, *TCF7*, *LEF1* and *SELL*, cytotoxicity scores were calculated using the following genes: *GZMA*, *GZMB*, *GZMK*, *GNLY*, *PRF1*, *IFNG*, *NKG7* and *IL2*, and exhaustion scores were calculated using the following genes: *LAG3*, *TIGIT*, *CTLA4*, *PDCD1* and *HAVCR2*.^41^ The memory signature genes were collected from CellMarker database.^86^

### 4.18 Proportion‐based survival analysis

Cell type relative abundance was obtained by running CIBERSORTx^87^ on RNAseq data of leukaemia samples. First, the single‐cell raw counts matrix of BM mononuclear cells with T cells split into three subtypes were fed into CIBERSORTx, building a gene signature able to discriminate the cell types or subtypes. Second, we collected survival tables of TCGA‐LAML and BROAD‐CLL cohorts from cBioPortal^88^ and ran survival analysis stratifying patients with ‘high’ and ‘low’ infiltration of cell type or subtypes.

### 4.19 eNet enhancer network complexity analysis

To explore the gene expression regulation associated with transplantation strategy, we applied eNet algorithm^89^ to cytotoxic‐related genes (*GZMA*, *GZMB*, *GZMK*, CX3CR1, *GNLY*, *PRF1*, *IFNG*, *NKG7*, *IKZF2* and *IL2*). Briefly, eNet identified putative enhancers within a ± 100 kb window around its transcription start site (TSS), then inferring potential enhancer interactions by computing chromatin co‐accessibility between enhancer pairs.^90^ The enhancer network was built as an undirected graph where nodes represent enhancers and edges represent the strength of enhancer interactions.

### 4.20 GNLY enhancer cluster analysis

CREs in the *GNLY* enhancer network constructed by eNet were preserved. CREs that overlapped with the gene promoter were defined as promoters, whereas the rest were defined as putative enhancers. CREs sequences were input into the FIMO^50^ motif scanning of the MEME Suite (https://meme‐suite.org/) with a motif database supplied by JASPAR2020.^82^ The FIMO output listing matching motif occurrences were filtered for matches with a *p* value < .0001. The list of statistically significant motif matches was further ranked by expression calculated by averaging the TF gene expression across all CD8^+^ T cells derived from the haplo+cord group.

## AUTHOR CONTRIBUTIONS

N. L., J. H. and Y. C. designed and supervised the research. P. C., X. C. and X. Y. were involved in patient inclusion and data acquisition. H. L., X. L. and Z. Zhang performed the analysis and interpretation of the clinical data. H. L., Z. Zhu and T. L. collected patient samples. M. Z., J. D., H. L., F. C. and Y. Y. performed single cell experiments and flow cytometry analysis. Z. Zhang, M. Z., R. T., H. Lin and M. T. performed the analysis and interpretation of multi‐omics data. Z. Zhang, H. L., M. Z., T. C. and J. H. wrote the manuscript with the input from other authors.

## CONFLICT OF INTEREST STATEMENT

The authors declare no conflicts of interest.

## FUNDING INFORMATION

This work was sponsored by Fujian Provincial Health Technology Project (2021ZD01005 to N. L.) and Fujian Province Science and Technology Major Special Project (2022YZ034016 to N. L.). This work was also supported by the National Natural Science Foundation of China (82470211 to N. L.; 92474104 and 32370586 to J. H.) and the Fundamental Research Funds for the Central Universities (20720230068 to J. H.); and the Wang Deyao Outstanding Graduate Scholarship Program of Xiamen University. At the same time, it was also sponsored by National Key R&D Program of China (2022YFC2502704 to X. L.) and Fujian provincial health technology project (2021GGA018 to X. L.).

## ETHICS STATEMENT

The study protocol was approved by the ethics committee of Fujian Medical University Union Hospital.

## CONSENT

Informed consent was obtained from enrolled patients before the study (Ethics Approval Number: 2021WSJK007). Participants provided written informed consent to publication of their clinical data.

## Supporting information



Supporting Information

Supporting Information

## Data Availability

All single‐cell sequencing raw data of this study are available in National Genomics Data Center (NGDC, https://ngdc.cncb.ac.cn/), under accession number: HRA002881 (shared URL: https://ngdc.cncb.ac.cn/gsa‐human/s/6lTWZZCV). The other resources used in this study are available from the corresponding authors upon reasonable request.
