# Supplementary material for: Single‐cell analysis reveals cytotoxic and memory CD8+ T cells associated with prolonged survival in relapsed/refractory leukaemia patients after haplo+cord haematopoietic stem cell transplantation
Source: Clin Transl Med. 2026 Feb 4;16(2):e70529. doi: 10.1002/ctm2.70529 (PMC12872979; doi:10.1002/ctm2.70529)
Supplement: Supplementary file 1 — Supporting Information [file CTM2-16-e70529-s002.docx]

# Supplementary figures


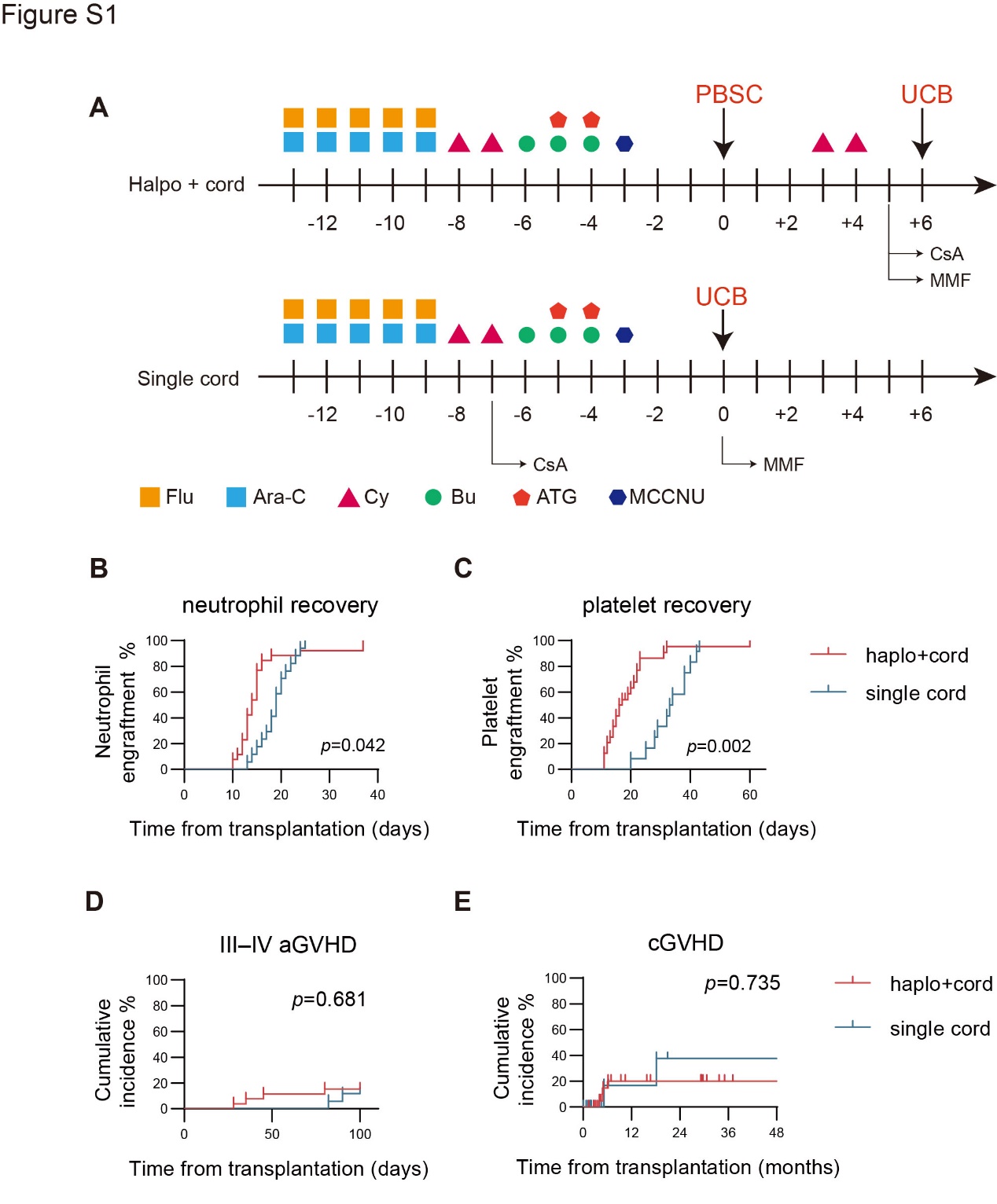


**Figure S1. Comparison of patients with R/R leukemia receiving haplo+cord or single cord HSCT.**

(**A**) Schematic diagram of haplo+cord and single cord HSCT. Flu, fludarabine; Ara-C, cytarabine; Cy, cyclophosphamide; Bu, busulfan; ATG, anti-thymocyte globulin; MMF, mycophenolate mofetil; CsA, cyclosporin A; PBSC, peripheral blood hematopoietic stem cells; UCB, umbilical cord blood stem cells.

(**B** - **E**) The Kaplan-Meier curves showing cumulative recovery of neutrophil (**B**) and platelet (**C**) as well as incidences of grade III–IV acute GVHD (**D**) and chronic GVHD (**E**) of 43 patients grouped by transplantation strategies. *P* values were calculated using the log-rank test.


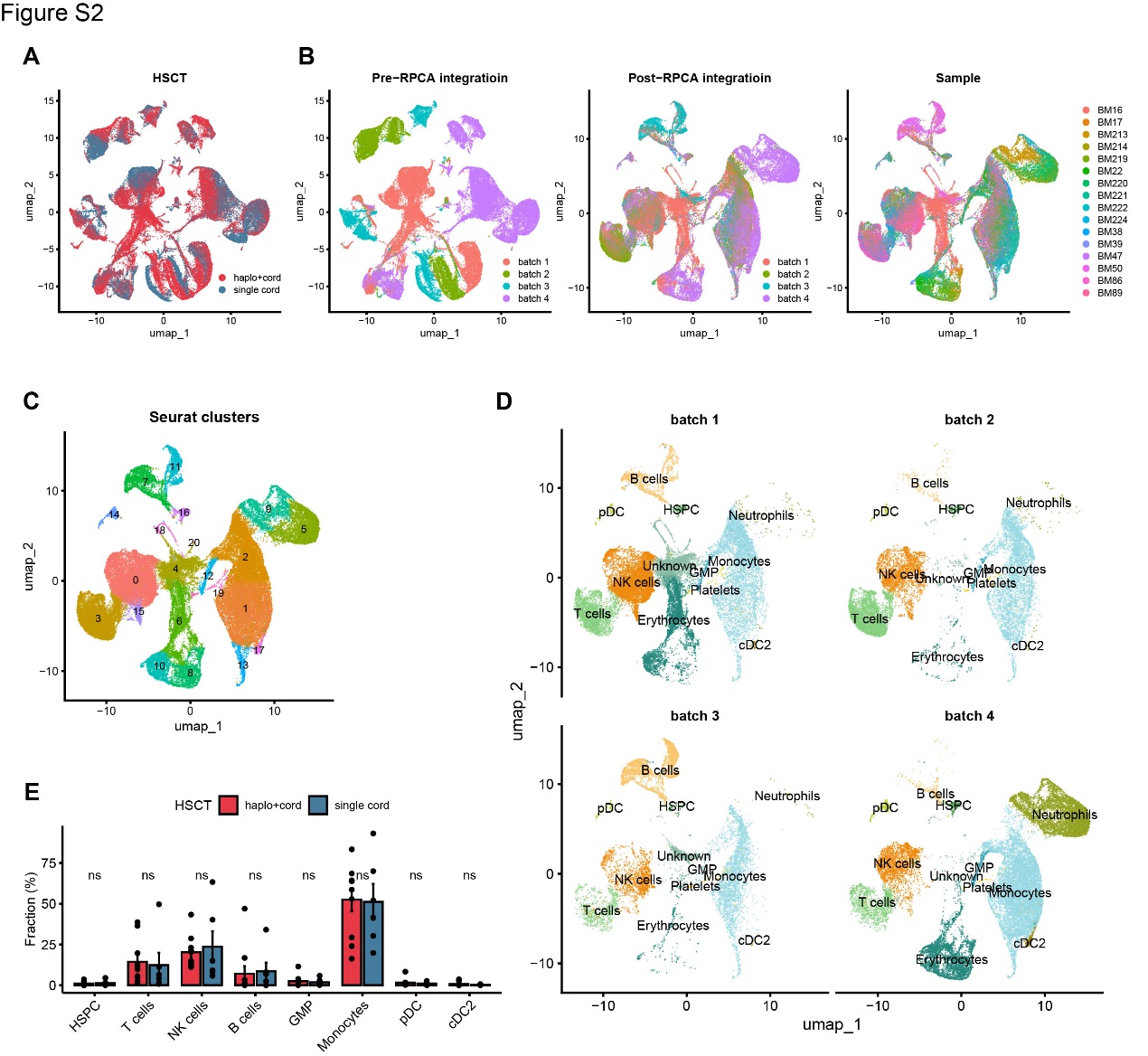


**Figure S2. Global immune landscapes and cell types in bone marrow after HSCT.**

(**A**) Uniform manifold approximation and projection (UMAP) of scRNA-seq data from 16 patients that received HSCT, colored by transplantation strategies.

(**B**) UMAP of scRNA-seq data showing embeddings pre-RPCA integration (left), post-integration (middle), and colored by individual sample (right). Batch effects associated with library construction time points are overcome.

(**C**) UMAP of scRNA-seq data from all cells colored by the Seurat clusters.

(**D**) UMAP of scRNA-seq data (shown in **Fig. 2A)**, split by experimental batches.

(**E**) Proportion of each major cell type in haplo+cord and single cord groups. Error bars are equal to the mean standard error (MSE) across individuals. Statistical analysis was performed using Wilcoxon signed-rank test.


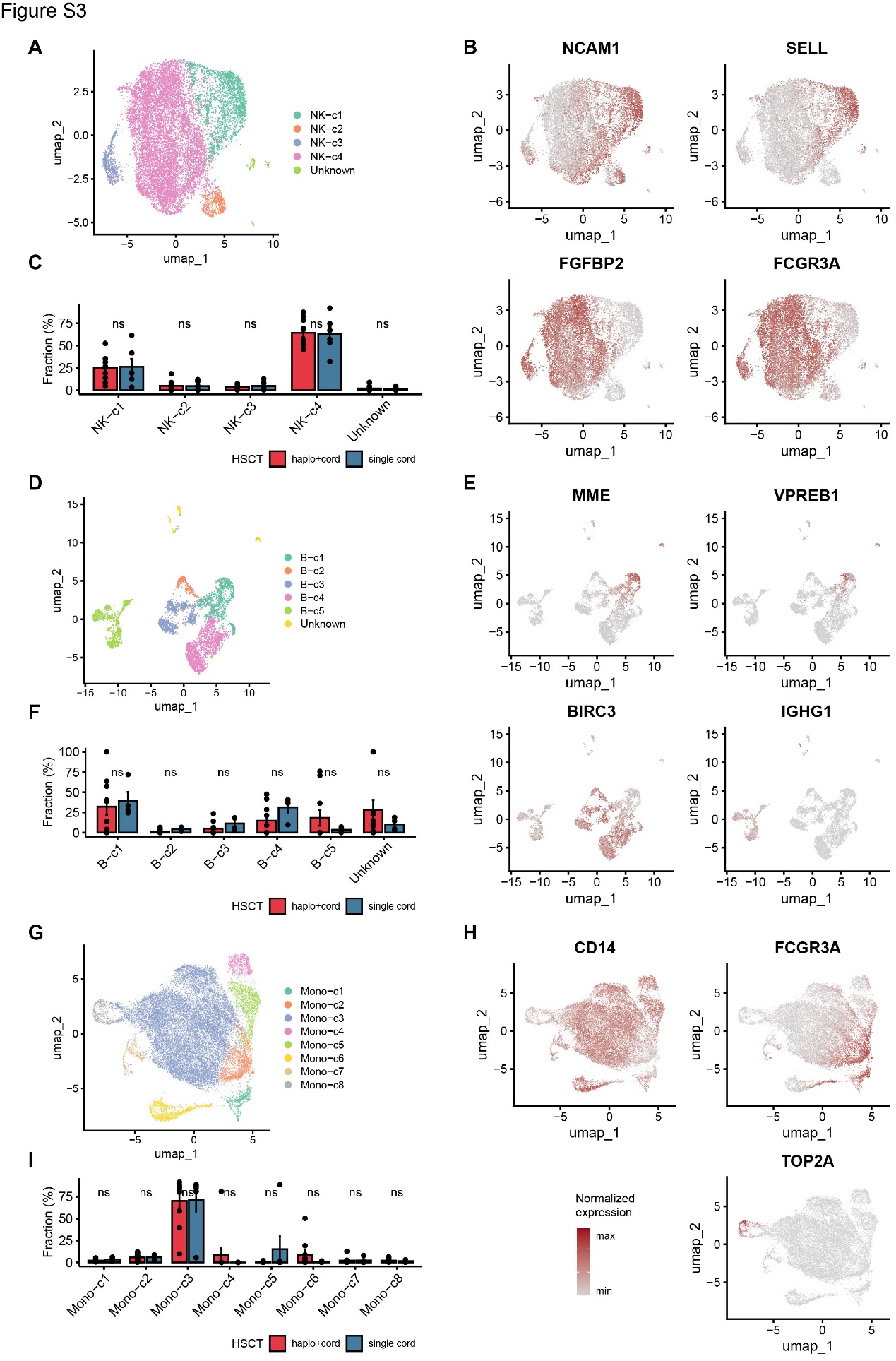


**Figure S3. Subclustering of NK cells, B cells, and monocytes.**

(**A** - **C**) Subclustering of NK cells. UMAP of 16,096 NK cells, identifying 4 subtypes, as indicated by different colors (**A**). Feature plot showing the expression of the known marker genes for NK cells (**B**). Proportion of each NK cell subtype in haplo+cord and single cord groups. Error bars are equal to the MSE across individuals. Statistical analysis was performed using Wilcoxon signed-rank test (**C**).

(**D** - **F**) Subclustering of B cells. UMAP of 6,075 B cells, identifying 5 subtypes, as indicated by different colors (**D**). Feature plot showing the expression of the known marker genes for B cells (**E**). Proportion of each B cell subtype in haplo+cord and single cord groups (**F**).

(**G** - **I**) Subclustering of monocytes. UMAP of 27,621 monocytes, identifying 8 subtypes, as indicated by different colors (**G**). Feature plot showing the expression of the known marker genes for monocytes (**H**). Proportion of each monocyte subtype in haplo+cord and single cord groups (**I**).


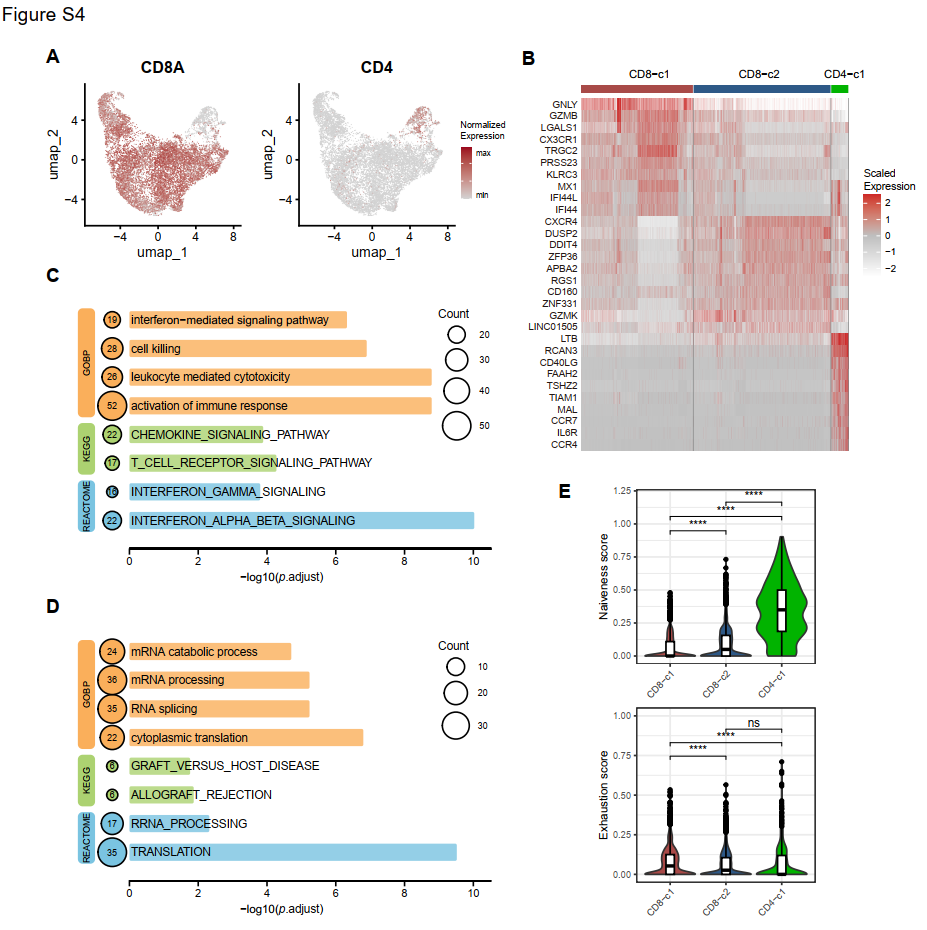


**Figure S4. CD8^+^ T cells reconstructed from different transplantation strategies in bone marrow.**

(**A**) Expression of T cell marker genes.

(**B**) Heatmap showing the expression of the marker genes in each T cell subtype.

(**C** and **D**) Representative pathways enriched in DEGs specifically expressed in CD8-c1 (**C**) and CD8-c2 (**D**) subtypes.

(**E**) Violin plots showing the distribution of naïveness scores and exhaustion scores in each T cell subtype. T cell functional scores were calculated using AUCell with gene sets related to their function-related genes. Statistical analysis was performed using Wilcoxon signed-rank test.


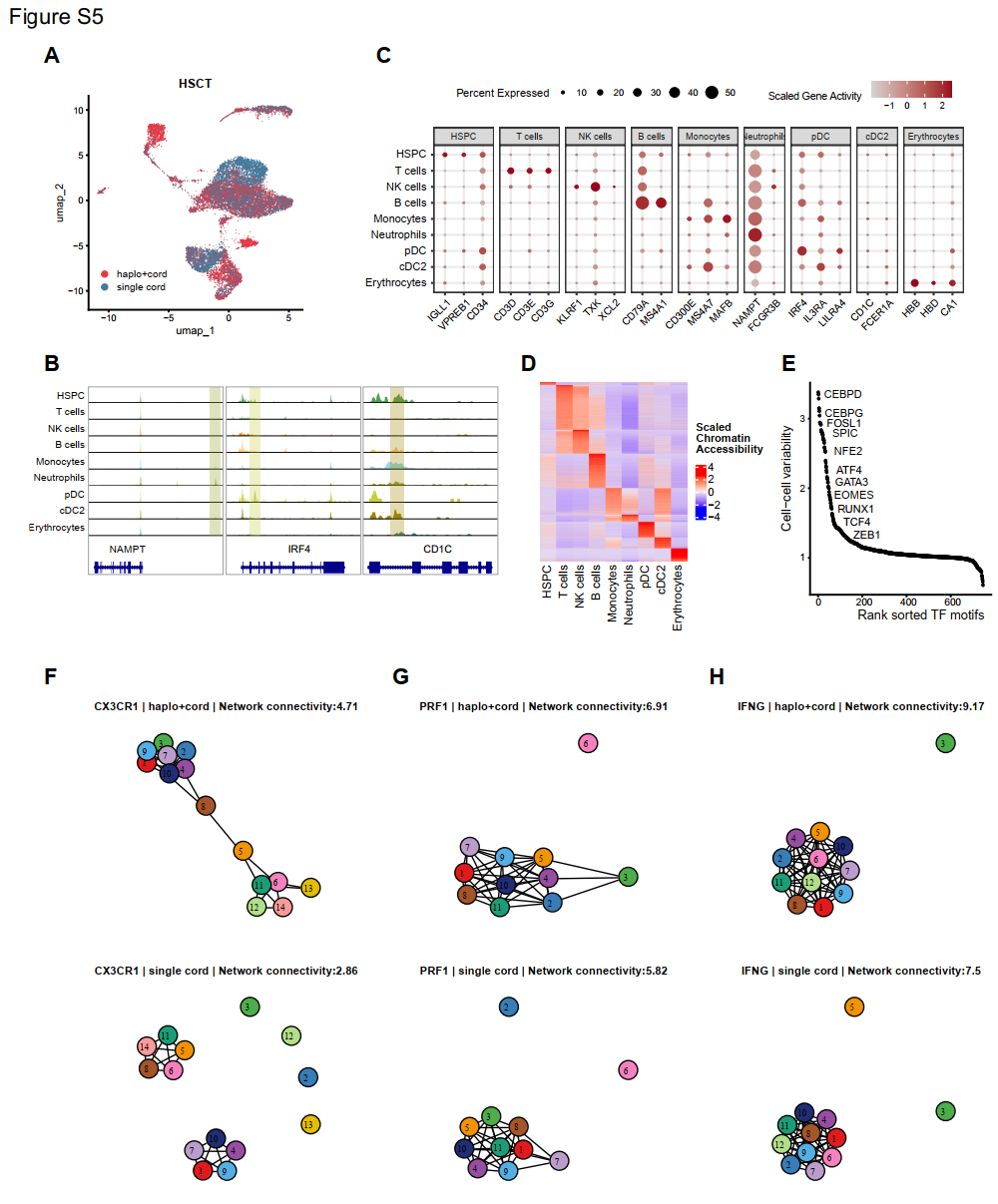


**Figure S5. Single-cell multi-omics analysis of bone marrow from patients receiving haplo+cord or single cord HSCT.**

(**A**) UMAP of scATAC-seq cells color-coded by transplantation strategies.

(**B**) Genome browser tracks showing aggregated chromatin accessibility of cells grouped by major cell types around the loci of marker genes, in addition to those shown in **Fig. 5B**.

(**C**) Inferred gene activity of known marker genes for the cell types defined in **Fig. 5A**.

(**D**) Heatmap showing the scaled chromatin accessibility of putative cis-regulatory elements (CREs) (rows) across 9 major cell types (columns).

(**E**) Variability analysis of TF motifs across all cells, in which they were ranked by cell-cell variability.

(**F** – **H**) eNet analysis revealed the transcriptional regulation network among CREs on the loci of representative cytotoxic-related genes *CX3CR1* (**F**), *PRF1* (**G**), and *IFNG* (**H**) in CD8^+^ T cells derived from haplo+cord and single cord groups. Each node represents a CRE, while each edge represents the potential interaction between two CREs predicted by eNet.


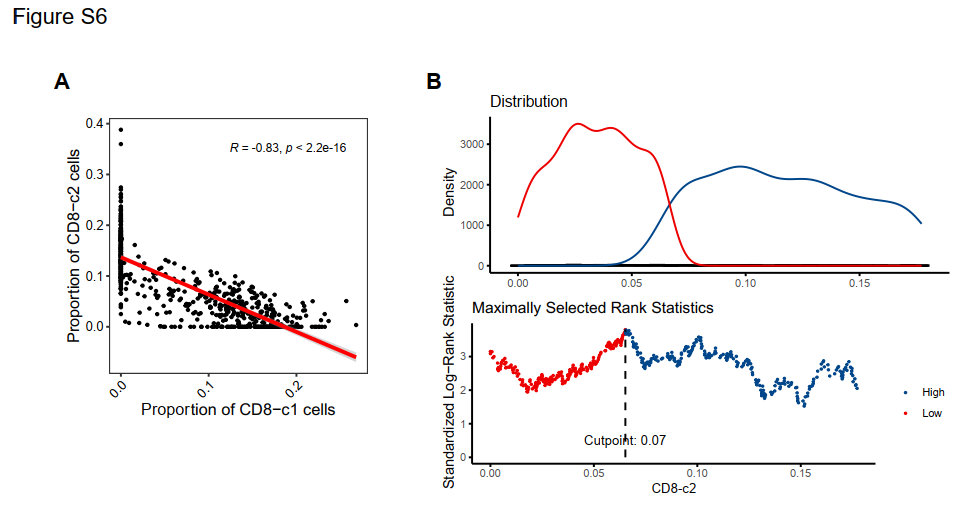


**Figure S6. The prognostic value of CD8+ T cell subtypes.**

(**A**) Scatter plot showing the correlation between cellular proportions of CD8-c1 and CD8-c2 subtypes across samples in BROAD-CLL.

(**B**) Distribution of the proportion of CD8-c2 across BROAD-CLL samples. The optimal cutpoint for stratifying high- and low- infiltration was obtained by using the Maximally Selected Rank Statistics.
